# Bleaching in vital teeth: Combined treatment vs in-office treatment

**DOI:** 10.4317/jced.56079

**Published:** 2019-08-01

**Authors:** Vicente Faus-Matoses, Iván Palau-Martínez, Jose Amengual-Lorenzo, Ignacio Faus-Matoses, Vicente-Jose Faus-Llácer

**Affiliations:** 1 Department of Stomatology, Universitat de València, Spain; 2 Dentist, Valencia, Comunidad Valenciana, Spain

## Abstract

**Background:**

The aim of this study was to verify that there will be greater whitening in teeth treated with combined bleaching than in those that have been applied a clinical one and to evaluate the efficiency of the clinical treatment, those cases in which it is not able, or it is not wanted, to carry out the home phase.

**Material and Methods:**

They were used 66 extracted anterior human teeth, which were divided into two study groups. On the one hand, the clinical group (ClG) consisted of 33 teeth, which were treated with a clinical guideline using 37.5% hydrogen peroxide in a single session of 4 applications of 8 minutes. On the other hand, the combinate group (CoG) consisted of 33 teeth, which were treated with a combined guideline, applying first a clinical treatment as in the ClG and, at home treatment with carbamide peroxide at 16% for 22 days, 90 minutes a day. The colour of the tooth was measured before and after each treatment and was made through an individualized whitening splint with a spectrophotometer.

**Results:**

The 66 teeth were bleached, showing an increase in luminosity, a drecrease in yellow and a shift towards the green colours, where b (yellow-blue axis) was the only variable with a statistically significant change (*p*<0.001). The CoG obtained a significantly higher absolute value (*p*<0.001) than the ClG, being 12.99 for the first one and 19.70 for the second one.

**Conclusions:**

Combined therapy bleached more than clinical one, but both techniques were effective. In addition, it is affirmed that the clinical could be an alternative in those cases in which it is not able, or it is not wanted, to carry out the home phase.

** Key words:**Carbamide peroxide, CIELab, combined guidelines, dental bleaching, hydrogen peroxide.

## Introduction

Dental bleaching is a relatively simple and conservative technique that makes possible to change dental colour by removing discolorations (1). The causes of those colour modifications are multiple and diverse, affecting vital and non-vital teeth in the same way (2).

Nowadays, the most used bleaching agents are hydrogen peroxide and carbamide peroxide, whose mechanism of action is basically related to the oxidation capacity of these bleaching agents on the molecules of the pigments responsible for the discoloration (3,4). Depending on the concentration of the bleaching agent, the product will have different indications. In vital teeth, there are different whitening guidelines: clinical, at home and combined treatment.

To obtain results that are less aggressive to the dental tissues and more durable with respect to the longevity of the bleaching, the current trend is to use a combination of techniques in-office and at-home (5,6). It has been shown that patients prefer those therapies which last less time (7), and combined treatment need more time than the clinical one.

The aim of this study was to verify that there will be greater whitening in teeth treated with combined bleaching than in those that have been applied a clinical one and to evaluate the efficiency of the clinical treatment, those cases in which it is not able, or it is not wanted, to carry out the home phase.

## Material and Methods

It has been used 66 human anterior teeth extracted in good condition and free of tartar, excluding those teeth with reconstructions that affected the vestibular face. They were divided, randomly, into two study groups. On the one hand, the clinical group (ClG) consisted of 33 teeth, which were treated with a clinical guideline using 37.5% hydrogen peroxide (Polaoffice + ® SDI). On the other hand, the combined group (CoG) consisted of 33 teeth, which were treated with a combined guideline, applying first a clinical treatment as in the ClG and, a home treatment with carbamide peroxide at 16% (Polanight ®. SDI).

Before taking the colour, the teeth were kept for two days immersed in physiological serum to acquire their natural tone. This protocol is done because if the tooth becomes dehydrated it becomes whiter and would cause us an error in the colour measurement (8).

All the teeth were placed in a model of plaster, in the shape of a horseshoe, to be able to make a splint, for taking colour, with a vacuum machine (Pro-form. Dental Resources Inc). This splint had got centre cavities on the vestibular face of each tooth, to make an accurate colour measurement. Prior to the bleaching treatment, the colour parameters of the space CIELab (L*, a*, b*) were recorded through the openings of the splint with the VitaEasyshade V® (Vita) spectrophotometer.

In both groups, the instructions for use specified by the manufacturer were followed in each product. On the one hand, in the ClG it was applied to the buccal surface of teeth Polaoffice+® in a single session of 4 applications of 8 minutes each one. On the other hand, the CoG were treated with a combined guideline, applying first a clinical treatment as in the ClG and at home treatment with carbamide peroxide at 16% (Polanight®) applied on the vestibular teeth surface for 22 days, 90 minutes a day.

Note, that the amount of product applied to the tooth is the detailed quantity by the manufacturer, which will be removed by brushing the tooth with water. Between the phases of treatment, the teeth were stored hydrated in a saline solution with a heat source that simulates the body temperature (37°C) in the oral medium.

In order to allow definitive stabilisation of the tooth colour, the last colour measurement was performed one week after concluding the treatment (1). The colour of the tooth was measured again with the VitaEasyshade V® spectrophotometer to see the colour parameters obtained at the end. Finally, the results were obtained from the colour difference formula CIELab (8). Furthermore, an absolute value was obtained, which would allow us to compare the colour change achieved in both techniques.

The results obtained by VitaEasyshade V® were analysed statistically using t-test with Welch correction. In all cases, *p* values < 0.05 were considered as statistically significant.

## Results

**Figure 1:**
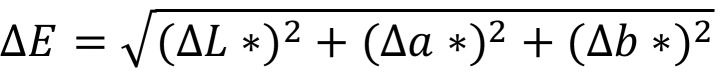
Formula.

To be able to compare both techniques it was used the formula CIELab (Fig. 1):

The absolute value that is obtained is the distance euclidea between two colour points in the CIE colour space L * a * b * ( Table 1).

**Table 1:**
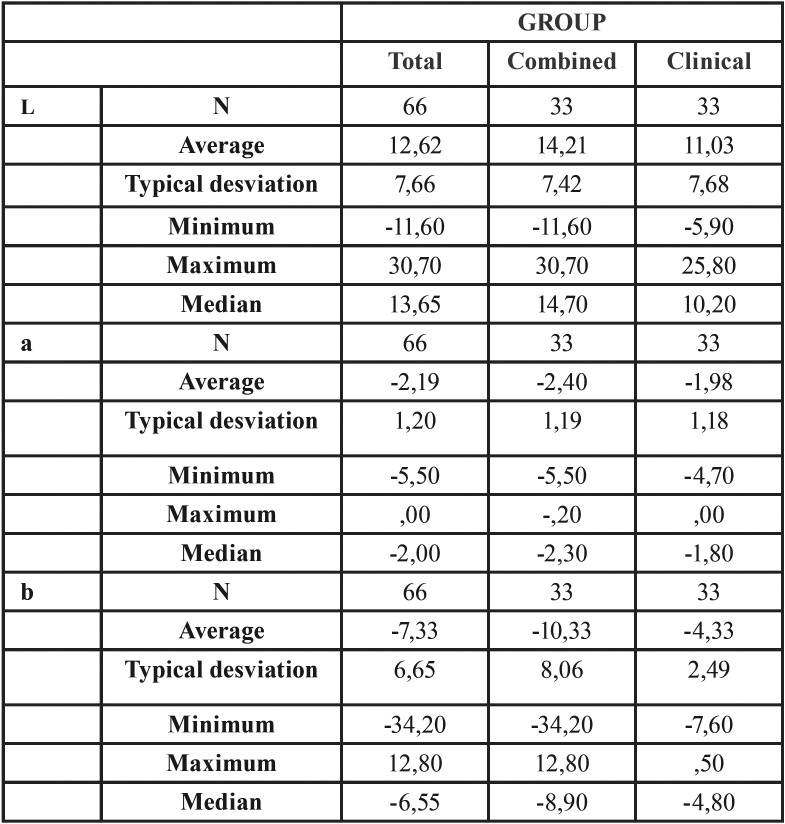
Descriptive analysis of the sample.

**Figure 2:**
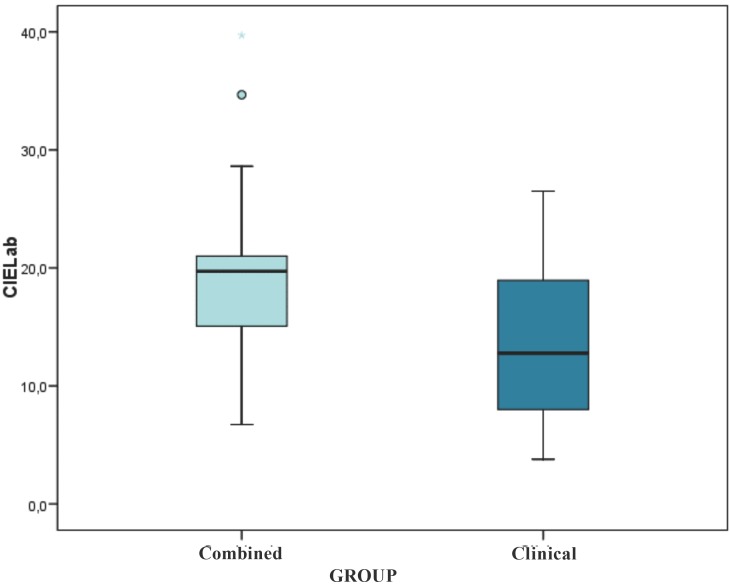
Absolute values obtained with the CIELab formula for each group.

Therefore, a value of 12.99 was obtained in the ClG. While, a value of 19.70 was obtained in the teeth bleached with a combined treatment (Fig. 2). The CoG exhibits a ∆E value significantly superior to the ClG (*p* <0.001).

**Figure 3:**
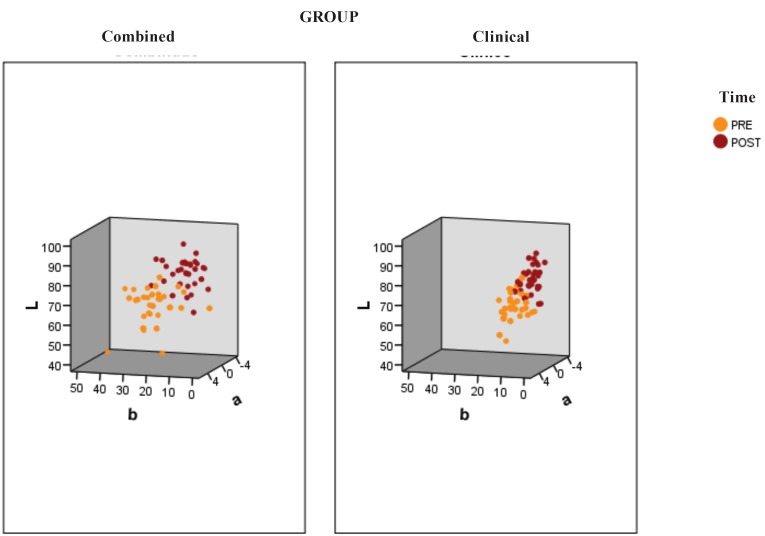
Displacement of the color variables in the CIELab formula: luminosity (L), red-green axis (a) and yellow-blue axis (b).

In the Figure 3 it could be observed and increasement of the brightness (L), a movement in the red-green axis (a) towards green tone, and a yellow decrease in the yellow-blue axis (b) towards the midpoint of the axis. It means that within the three-dimensional point cloud, L * moves up, while a * tends towards green and b* approaches to the center of the axis. Furthemore, as it can be seen, the ClG has less modification than CoG.

On the other hand, analysing each group separately, both shown a significant (*p*<0,001) effectiveness treatment and an effectiveness treatment analysing each component (L, a*, b*).

## Discussion

As we could see in the results, the combined group exhibits ΔE values significantly superior to the clinical group (*p* <0.001). It can be affirmed that the ΔE value is higher in the CoG because of the elevation of the L* component, and the biggest modification of a* and b* components, compared to the clinical treatment.

In the Rodrigues and cols. article comparing the combined whitening pattern versus the clinical one (9) and evaluated the effect of associating at-home and in-office bleaching procedures on tooth sensitivity and bleaching effectiveness. In conclusion, after one in-office bleaching session, there was no difference in bleaching effectiveness between performing a second in-office session and associating it with 1-week at-home bleaching. In this study, this one was done in forty patients subjected to one session of in-office bleaching with 38% peroxide hydrogen. Subsequently, the patients were randomly allocated to receive a second session of in-office bleaching and to use a tray containing 10% carbamide peroxide delivered only during 7 consecutive days. Nevertheless, colour was assessed by a spectrophotometer as the present study, but Rodrigues and cols. also used a colour match with the Vita Classical and Bleach guide scales as other authors do (5,10,15). Also, Rezende and cols. (10), they used a technique combined with 35% hydrogen peroxide in one session and then 10% carbamide peroxide for two weeks 2 hours a day. In this study, patients had to present incisors with a darker colour to A2. Finally, measuring with a shade guide they saw how at the beginning only with the combined technique a variation of 1.9 shades was obtained while at the end they obtained 3.2 shades after using at home. Despite this difference, the author highlights how patients usually demand treatments with more rapid effects and that the at home schedule is very slow.

Furthermore, Delipieri and cols. (5) measured with a subjective system of shades explained that combination of in-office and take-home bleaching would produce a tooth colour modification greater than 5 shades on the shade guide arranged in value order. That it means that combined guideline bleached teeth as the present study achieved. Radz and cols. (13) also demonstrated the effectiveness of combined treatment achieving a modification of 11.1 shades. This study examined nine subjects with baseline shades of A3.5 to A4 on four to six of their maxillary anterior teeth with the VITA Classical (VC) shade guide. All subjects received one session of in-office whitening (Philips Zoom WhiteSpeed), 3 weeks of at-home tray whitening (Zoom NiteWhite).

On the other hand, Giachette and cols. (11) demonstrated the effectiveness of in-office whitening using 38% hydrogen peroxide, but none of the L*, a* and b* components showed no statistical difference. Conversely, in the present study, the b* component had a statistical difference before and after bleaching. Similarly, other authors (13,14) confirmed the effectiveness of hydrogen peroxide in whitening in office. Specifically, Matis and cols. (12) evaluated the effectiveness of Pola Office +, confirming its effectiveness use and its colour stability over time. Monteiro and cols. (14) used 35% hydrogen peroxide gel in office with light-curing in 40 patients using a splint for taking the colour, as the present study did. After 15 days they saw how bleaching was effective and the only variable with a statistically significant change was the b* component. Also, Monteiro and cols. (14) explains how this in-office technique achieves rapid whitening.

Similarly, in the study by Marson and cols. (15), they studied the stabilization of colour with different in-office whitening techniques. Thus, they saw how the technique used in the present study had a statistically significant change reaching an ∆E of 12. In addition, they explain how in the first 24 hours a very large increase in L * is reached, which decreases progressively until it stabilizes in the first 15 days.

## Conclusions

As a result, we can conclude this study by saying:

Both techniques were effective.

There is a bleaching difference between both groups.

Combined therapy bleached more than clinical one,

There is not as much change as initially expected.

It is affirmed that the clinical treatment is viable for patients who for personal reasons do not want or can perform the phase of home treatment.

More studies are necessary to establish the duration of the results obtained with both techniques.
